# Characterization of Driver Mutations in Anaplastic Thyroid Carcinoma Identifies *RAS* and *PIK3CA* Mutations as Negative Survival Predictors

**DOI:** 10.3390/cancers12071973

**Published:** 2020-07-20

**Authors:** Wei-An Lai, Chih-Yi Liu, Shih-Yao Lin, Chien-Chin Chen, Jen-Fan Hang

**Affiliations:** 1 Department of Pathology and Laboratory Medicine, Taipei Veterans General Hospital, Taipei 112, Taiwan; walai@vghtpe.gov.tw (W.-A.L.); sylin20@vghtpe.gov.tw (S.-Y.L.); 2 Division of Pathology, Sijhih Cathay General Hospital, New Taipei City 221, Taiwan; cyliu@cgh.org.tw; 3 Department of Pathology, Far Eastern Memorial Hospital, New Taipei City 220, Taiwan; 4 Department of Pathology, Ditmanson Medical Foundation Chia-Yi Christian Hospital, Chiayi 600, Taiwan; hlmarkc@gmail.com; 5 Department of Cosmetic Science, Chia Nan University of Pharmacy and Science, Tainan 717, Taiwan; 6 National Yang-Ming University School of Medicine, Taipei 112, Taiwan

**Keywords:** anaplastic thyroid carcinoma, *RAS*, *BRAF*^V600E^, *PIK3CA*, *TERT* promoter, *TP53*

## Abstract

Anaplastic thyroid carcinoma (ATC) is rare but highly aggressive. We investigated the association of selected driver mutations, including *BRAF*, *RAS,* *PIK3CA*, *TERT* promoter, *TP53*, *POLE*, and mismatch repair deficiency (MMR-D) with the clinicopathological features of ATC to identify prognostic and predictive biomarkers. Thirty-nine retrospective cases from pathology archives were enrolled for clinicopathology analysis and immunohistochemistry, and 27 cases had sufficient specimens for further molecular testing using targeted next-generation sequencing and mass spectrometry. *BRAF^V600E^* and *RAS* mutations were identified in 25.9% and 40.7% of ATC, respectively. *BRAF^V600E^* mutation was significantly associated with coexisting papillary thyroid carcinoma (*p* = 0.009) and *RAS* mutations with female gender (*p* = 0.012). In univariant analysis, the non-*BRAF/RAS* tumors were significantly associated with the presence of a sarcomatoid pattern (*p* = 0.045). *PIK3CA*, *TERT* promoter, and *TP53* mutations were identified in 14.8%, 81.5%, and 70.4% of cases, respectively. No MMR-D or *POLE* mutations were detected. In survival analyses, *RAS* and *PIK3CA* mutations were significantly associated with inferior outcomes (*p* = 0.03 and *p* = 0.006, respectively). In conclusion, driver mutations in ATC are associated with distinct clinicopathological features. *RAS* and *PIK3CA* mutations were negative predictors for patient survival. Emerging therapeutic agents targeting BRAF, RAS, and PI3 kinase may benefit a substantial proportion of ATC patients.

## 1. Introduction

Thyroid carcinoma is the most common malignancy of the endocrine system [1]. Anaplastic thyroid carcinoma (ATC) is an aggressive tumor comprising 1%~2% of thyroid carcinoma [2]. Most patients present with advanced disease at the initial clinical encounter, and up to 90% of them die within a year after diagnosis [3,4]. ATC is managed by a multi-modality treatment approach combining surgical resection and chemoradiation. However, the effectiveness of the current treatment approach is limited [2]. As such, identification of actionable treatment targets, as well as biological predictors for clinical behavior in ATCs, is of value to improve clinical outcomes.

Genomic-scale analyses using next-generation sequencing have unraveled close genetic and phenotypic associations in well-differentiated thyroid carcinoma (WDTC). *RAS*-activating mutations and *BRAF*^V600E^ mutation are the most common driver mutations in WDTC [5,6]. *RAS* mutations are associated with follicular patterned thyroid tumors including follicular adenoma, follicular carcinoma (FTC), and follicular variant of papillary thyroid carcinoma (PTC) [5]. These follicular patterned tumors share a *RAS*-driven transcriptome [5]. In contrast, *BRAF*^V600E^ mutation is highly associated with classical PTC and tall cell variant of PTC. The *BRAF*-driven PTC has lower expression of thyroid differentiation genes, a higher risk of tumor recurrence, and strong MAP kinase signaling activation as opposed to activation of both MAP kinase and PI3 kinase pathways in *RAS*-driven PTC [7]. *RAS* and *BRAF*^V600E^ mutations were also commonly mutated genes in ATC [8,9,10,11,12,13,14,15,16,17]. Studies indicated that *RAS*-driven and *BRAF*-driven ATC share homogenous transcriptome with an undifferentiated thyroid gene expression profile [9,18].

*TERT* promoter and *TP53* were genomic hallmarks of ATC and less frequently detected in WDTC [19]. Additionally, mutations of PI3 kinase-Akt pathway-related genes were also identified in ATC [2,6,20,21]. Moreover, an increase of somatic mutation in ATC as compared to WDTC has been reported [15,18,22]. Defects in DNA mismatch repair mechanism or *DNA polymerase epsilon* (*POLE*) mutation could contribute to hypermutation in tumors and have therapeutic implications for the emerging immunotherapies [23]. It has been proposed that these molecular changes are acquired during tumor de-differentiation and contribute to anaplastic transformation in thyroid carcinoma [19]. However, the phenotypic association with the aforementioned genetic alterations has not been well delineated in ATC. In this study, we aimed to characterize these selected driver mutations and their associated clinicopathological features in ATC.

## 2. Results

### 2.1. Clinicopathological Features of ATC

**Figure 1:**
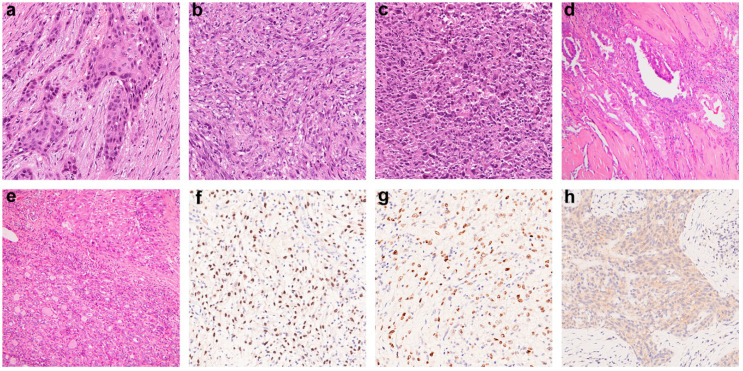
Anaplastic thyroid carcinoma. Common morphologic patterns include epithelial (**a**), sarcomatoid (**b**), and giant cell (**c**) pattern (H&E, 200×). Papillary carcinoma (**d**) and follicular carcinoma (**e**) coexist with the anaplastic component in an intermingled arrangement or with a sharp border (H&E, 100×). Cases positive for PAX8 (**f**), TTF-1 (**g**), and BRAF VE1 (**h**) immunostains are shown (200×).

We assembled a cohort of 39 ATC cases. The clinicopathological features are summarized in Table 1. The median age was 75 years. Female patients comprised 51.3% of the cases. More than half (55.6%) were stage IVC disease at initial presentation. Most patients underwent total or subtotal thyroidectomy (61.5% and 23.1%), while 15.4% of cases had biopsy only. Up to 51.3% of the patients received additional therapies, including external radiation (25%), chemotherapy (24.1%), radioiodine (21.4%), and targeted therapy (15.1%). Histologically, epithelial, sarcomatoid, and giant cell are well-described morphologic patterns in ATC (Figure 1a–c). Twelve (30.8%) cases showed more than one histologic pattern. In our cohort, the epithelial pattern was the most frequent (71.8%), followed by sarcomatoid (43.6%) and giant cell (12.8%). A coexisting well-differentiated thyroid carcinoma component was identified in 33.3% of cases, including 10 PTC and 3 FTC (Figure 1d,e). Immunohistochemical stains were utilized to detect thyroid-lineage marker expression and *BRAF*^V600E^ mutation (Figure 1f–h). PAX8 expressed in 56.4% and TTF-1 in 5.1% of the cases (Table 2). BRAF VE1 staining was positive in 25.6% of all cases. Coexisting PTC was identified in 6/10 (60%) of the BRAF VE1-positive ATC and 4/29 (13.8%) of wild-type ATC. Coexisting PTC was significantly associated with *BRAF*^V600E^ mutation (*p* = 0.009).

### 2.2. Molecular Profiles of ATC

**Figure 2:**
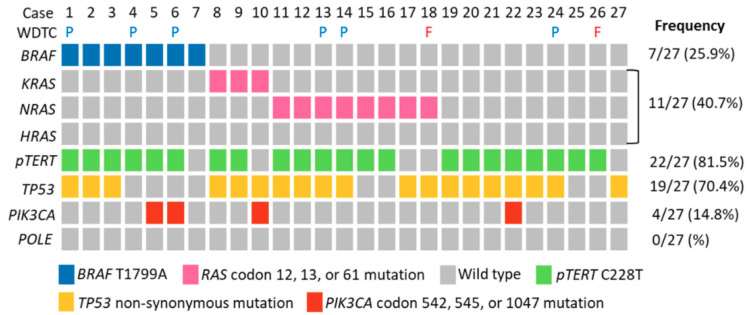
Summary of the mutational profile of the 27 cases of anaplastic thyroid carcinoma analyzed by next-generation sequencing and mass spectrometry. WDTC, coexisting well-differentiated thyroid carcinoma; P, papillary carcinoma; F, follicular carcinoma; *pTERT*, *TERT* promoter.

Twenty-seven ATC cases with sufficient formalin-fixed paraffin-embedded (FFPE) materials for DNA extraction were subjected to targeted next-generation sequencing (NGS) and mass spectrometry, and the results are summarized (Figure 2, Supplementary Data 1 and Data 2). *BRAF*^V600E^ (c.T1799A) was identified in 25.9% of the cases. All ATC with *BRAF*^V600E^ mutation detected by NGS were positive for the BRAF VE1 immunohistochemistry. *RAS*-activating mutations at codon 12, 13, and 61 were analyzed. We found overall *RAS* mutations in 40.7% of the cases, and among the 11 cases, 8 were found in *N-RAS* (Q61R in 6, Q61K in 1, and G12D in 1) and 3 in *K-RAS* (Q61R in 2 and G12A in 1). *H-RAS* mutation was not identified. To investigate the potential association between driver mutations and clinicopathological features, the 27 cases were stratified into three molecular groups: *BRAF*-mutated, *RAS*-mutated, and non-*BRAF/RAS* group (Table 3). We found that *RAS* mutation was associated with female gender (90.9% versus 44.4% and 28.6% in non-*BRAF/RAS* and *BRAF* groups respectively, *p* = 0.012). The non-*BRAF/RAS* group was associated with the presence of a sarcomatoid component morphologically (77.8% versus 45.5% in *RAS* and 14.3% in *BRAF* groups, respectively, *p* = 0.045). Association between *RAS* mutations and female gender remains significant (odds ratio 20.31, *p* = 0.022, supplementary Table S1) in multivariate analysis, while the non-*BRAF/RAS* group becomes marginally associated with the presence of a sarcomatoid pattern (odds ratio 23.17, *p* = 0.089, Supplementary Table S2). *TERT* promoter C288T mutation was present in 81.5% of cases, while C250T was not detected in any of the cases. *TP53* missense mutation was found in 70.4% of ATC. *PIK3CA* mutation was present in 14.8% of ATC, including one E542K, one E545K, and two H1047R mutations. Mismatch repair deficiency and *POLE* exonuclease domain mutation potentially led to a hypermutation phenotype in tumors. We found that mismatch repair (MMR) protein expression was retained in all of the cases (Table 2). We did not identify mutations in the exonuclease domain of *POLE* in any of the ATC.

### 2.3. Survival Analysis of ATC Cohort

**Figure 3:**
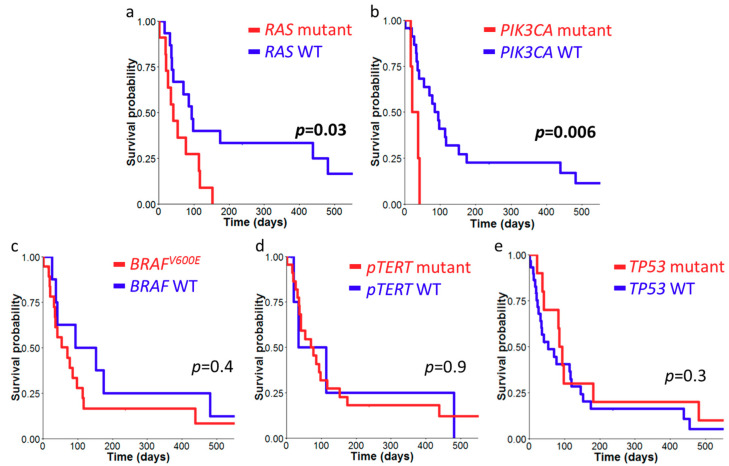
Survival analysis of anaplastic thyroid carcinoma (ATC). Kaplan–Meier survival analysis was performed with cases stratified by *RAS* mutations (**a**), *PIK3CA* exon 2 and exon 9 mutations (**b**), *BRAF*^V600E^ mutation (**c**), *TERT* promoter mutation (**d**), and *TP53* non-synonymous mutations (**e**). Survival differences were analyzed by log-rank test with significance at *p* value < 0.05 (bold type). WT, wild-type; *pTERT*, *TERT* promoter.

Follow-up data of all cases was obtained, and the median survival interval was 54 days (1–1079). We constructed Kaplan–Meier survival curves with stratification on molecular alternations including *BRAF*^V600E^, *RAS*, *TERT* promoter, *TP53,* and *PIK3CA* mutations (Figure 3). *RAS* and *PIK3CA* mutations were significantly associated with an inferior survival (median 94 versus 41 days, *p* = 0.03 and median 94 versus 29 days, *p* = 0.006 respectively). None of the other mutations correlate with patient survival.

## 3. Discussion

In the current study, we analyzed a cohort of ATC and identified *RAS* mutations in 40.7% and *BRAF*^V600E^ mutation in 25.9% of ATC. The two mutations were mutually exclusive and detected in up to 70% of ATC. ATC stratified by *BRAF*^V600E^ and *RAS* mutations showed distinct clinicopathological features. *BRAF*^V600E^ mutation was significantly associated with coexisting PTC. *RAS*-mutated ATC was positively associated with female gender and an inferior survival outcome. Non-*RAS/BRAF* tumors tended to show a sarcomatoid morphological pattern. *PIK3CA* mutations were found in 14.8% of ATC and were also identified as a negative prognostic indicator. *TERT* promoter and *TP53* mutations were frequent mutations in ATC, identified in 81.5% and 70.4% of the tumors respectively. MMR deficiency and *POLE* exonuclease domain mutation were not detected in our cohort. Except for *RAS* and *PIK3CA* mutations, none of the other molecular changes investigated in this study showed prognostic significance.

Genetic alternation rates of selected genes in ATC reported in the literature are summarized in Table 4. *RAS* mutation rate in ATC was reported ranging from 18% to 44.4%. In our cohort, *N-RAS* mutation was the most frequent (eight cases, 29.6%) among the three *RAS* subtypes, concordant with findings in previous reports [9,13,15,16]. *K-RAS* mutation was present in three cases (11.1%), while *H-RAS* mutation was not detected. Mutations at codon 61 were the most common, followed by codon 12. *RAS*-mutated ATC emerged with distinct clinicopathological features. *RAS* mutations were strongly associated with female gender in both univariate and multivariate analyses (*p* = 0.012 and 0.022) and showed significantly poor survival outcomes (*p* = 0.03). It has been reported that the co-occurrence of *RAS* and *TERT* promoter mutations was associated with an inferior survival outcome [8,18]. Our study was the first one to demonstrate that *RAS* mutations alone predicted a significantly poor outcome. On the other hand, *BRAF*^V600E^ mutation rate in ATC was reported ranging from 12.8% to 56% (Table 4). In one Korean report where PTC is prevalent, the *BRAF*^V600E^ mutation in ATC was up to 91% [14]. In our cohort, *BRAF*^V600E^ mutation was also strongly associated with a coexisting PTC component (*p* = 0.009). In the 10 ATC with coexisting PTC, up to 60% harbored *BRAF*^V600E^ mutation. Therapies targeting *BRAF*^V600E^ are being utilized for the treatment of ATC. In *BRAF*^V600E^-mutated ATC, combination treatment with Dabrafenib and Trametinib has been proven robust as an adjuvant therapy to improve clinical outcomes and a neoadjuvant treatment to facilitate complete surgical resection for local control [24,25]. Direct inhibitors of RAS have not yet achieved clinical success. Recently, small molecules targeting KRAS G12C have entered phase 1 and 2 clinical trials for the treatment of colorectal carcinoma [26]. Targeting RAS may become a potential strategy in the management of advanced malignancy. Among the 27 cases subjected to NGS studies, nine cases were absent of either *RAS* or *BRAF*^V600E^ mutations. Whether these tumors harbor other rare targetable driver fusion events found in WDTC, such as *RET-PTC*, *PAX8-PPARG*, *NTRK,* and *ALK* rearrangement, requires further analysis [27].

Mutation rates of *PIK3CA*, *TERT* promoter, and *TP53* are higher in ATC as compared to WDTC [19]. These gene mutations often co-occur with *BRAF*^V600E^ and *RAS* mutations and are thought to be acquired during thyroid cancer de-differentiation [19]. In the 18 cases with the driver *BRAF*^V600E^ or *RAS* mutations, only one was absent of all *PIK3CA*, *TERT* promoter, and *TP53* mutations. *PIK3CA* mutation rates were reported ranging from 0% to 44% in the literature (Table 4). We identified *PIK3CA* mutations in 14.8% of our cohort, with two cases co-occurring with *BRAF*^V600E^ mutation and one with *RAS* mutation. Moreover, we identified *PIK3CA* mutation significantly associated with an inferior survival outcome (*p* = 0.006). Duan et al. also reported *PIK3CA* as a negative predictor of patient survival in ATC, and co-occurrence of *PIK3CA* and *TERT* promoter mutations had a higher risk of mortality than patients with only one of either mutation [10]. PI3 kinase pathway and MAP kinase pathway are divergent pathways downstream of RAS signaling. Pre-clinical studies had identified combined treatment with MEK and PI3 kinase inhibitors synergized to target *BRAF*^V600E^ and *PIK3CA* double-mutant ATC cell lines and genetically engineered mice [28]. PI3 kinase inhibitors have also been evaluated in clinical trials for breast, colorectal, and lung cancers [29]. Whether targeting PI3 kinase serves as an effective treatment strategy for ATC remains to be elucidated. Mutations of *TERT* promoter and *TP53* are the genetic hallmark of ATC [2,12,20]. In ATC, the rate of *TERT* promoter and *TP53*-inactivating mutations ranged from 32% to 75% and 25% to 65.6%, respectively (Table 4). Moreover, *TERT* promoter mutation had been recurrently reported to associate with shorter survival [8,10,18]. Xu et al. and Duan et al. both showed that *TERT* promoter mutation alone served as a negative prognostic indicator [8,10]. Xu et al. further found that patient outcome was particularly grave in tumors with co-occurrence of *TERT* promoter mutation with *RAS* or *BRAF*^V600E^ mutation [8]. Yet no prognostic effect of *TERT* promoter mutation could be observed in our analysis. This could be attributed to a high mutation rate of *TERT* promoter in our cohort.

Previous studies reported an increase in somatic mutation numbers in ATC [15,18]. In colorectal and endometrial carcinoma, it was well demonstrated that MMR-D or *POLE* exonuclease domain mutation could lead to a marked increase of somatic mutations in tumors and predict response to anti-PD-L1 treatment [30,31,32]. Prior studies demonstrated MMR-D in up to 13.6% of ATC [8,15,22,33], and *POLE* missense mutation in 4% and 9% (Table 4). The mutation sites of *POLE* were not specified in previous studies. In our cohort, all ATC had preserved MMR protein expression, and we did not detect any non-synonymous mutations specifically in the *POLE* exonuclease domain. Our findings along with previous reports suggested that MMR-D and *POLE* exonuclease domain mutations were rare in ATC and had limited implications regarding anti-PD-L1 therapy.

In this study, we identified distinct clinicopathological features in ATC stratified by *BRAF*^V600E^ and *RAS* mutations. *BRAF*^V600E^ mutation was significantly associated with coexisting PTC, while *RAS*-mutated tumors were strongly associated with female gender. Of note, *RAS* and *PIK3CA* mutations were significantly associated with worse prognostic outcomes in ATC. Genetic hallmarks including *TERT* promoter and *TP53* mutations were prevalent, while MMR-D or *POLE* exonuclease domain mutations were not identified in our ATC cohort. The molecular profile of these selected driver mutations has clinical implications regarding the emerging targeted therapies in this particularly aggressive cancer.

**Table 4:** Literature review of molecular alternations in anaplastic thyroid carcinoma.

References	Case No.	*BRAF^V600E^*	*RAS*	*TP53*	*TERT*	*PIK3CA*	*POLE*	MMR Alternation ^b^
**Current Study**	27	25.9%	40.7%	70.4%	81.5%	14.8%	0%	0%
Xu 2020 [8] ^a^	102	43.1%	22%	63%	75%	18%	4%	8%
Khan 2019 [16]	90	32%	26%	65.6%	32%	12.2%	na	na
Duan 2019 [10]	25	56%	24%	60%	56%	44%	na	na
Yoo 2019 [9]	27	40.1%	44.4%	48.1%	55.6%	11.1%	na	na
Ravi 2019 [33]	11	18%	18%	55%	36%	18%	9%	9%
Chen 2018 [17]	12	25%	33%	25%	na	0%	na	na
Bonhomme 2017 [13]	94	12.8%	42.6%	54.4%	54%	6.4%	na	na
Kunstman 2015 [15]	22	27.3%	27.3%	27.3%	na	9.1%	na	13.6%

^a^ Cases from Landa et al. 2016 [18] and Pozdeyev et al. 2018 [34] were included in Xu 2020; ^b^ MMR alternations include MMR deficiency detected by immunohistochemistry in this study as well as mutations of the MMR genes detected in Xu 2020, Ravi 2019, and Kunstman 2015; MMR, mismatch repair; na, not analyzed.

## 4. Materials and Methods

### 4.1. Case Selection

This study was carried out in accordance to the Declaration of Helsinki and approved by the Institutional Review Board of Taipei Veterans General hospital, which granted exemption of informed consent for tissue procurement through the Biobank of Taipei Veterans General Hospital after an unlinked anonymous process (IRB no.: 2018-06-008BC, June 7th, 2019; Biobank no.: 10707). A total of 39 ATC cases from four participating hospitals in the period between 1997 and 2018 were enrolled. All pathology slides were reviewed, and the diagnosis of ATC in each case was confirmed according to the 2017 WHO Classification of Tumors of Endocrine Organs [35]. Histologic patterns, including epithelial, giant cell, and sarcomatoid, as well as the presence of coexisting differentiated carcinoma component, were recorded. Clinical information including age, gender, stage at presentation, specimen obtaining procedure, treatment modality, and follow-up information was obtained from the biobank database.

### 4.2. Construction of Tissue Microarray and Immunohistochemistry

For each case, representative formalin-fixed paraffin-embedded (FFPE) ATC tissue was selected, and two 2-mm cores were obtained for tissue microarray construction. Four-μm tissue microarray sections were used for H&E and immunohistochemistry. Immunohistochemical stains for PAX8 (1:400 dilution; clone 10336-1-AP; Proteintech, Rosemont, IL), TTF-1 (1:300 dilution; clone 8G7G3/1, Dako GmbH, Jena, Germany), and BRAF VE1 (1:200 dilution; clone VE1; Spring Bioscience, Pleasanton, CA, USA) were performed on Leica Bond-Max autostainer (Leica Microsystems GmbH, Wetzlar, Germany). To investigate mismatch repair protein (MMR) deficiency, immunohistochemical stains for the four MMR proteins, including MSH2, MSH6, PMS1, and MLH2, were performed. Prediluted antibodies for MLH1 (clone M1), PMS2 (clone A16-4), MSH2 (clone G219-1129), and MSH6 (clone SP93) were applied on BenchMark Ultra slide stainer (Ventana Medical Systems, Tuscan, Italian). The automated program for immunohistochemistry included deparaffinization, antigen retrieval, incubation with primary antibody followed by secondary antibody, and chromogen visualization. For PAX8 and TTF-1, positive staining was defined as the presence of positive nuclear staining in more than 5% of ATC tumor cells. For BRAF VE1, positive staining was defined as diffuse cytoplasmic staining of moderate to strong intensity. Loss of MMR protein expression was defined as complete absence of nuclear staining in the entire tumor area with lymphocytes and vascular endothelial cells serving as positive internal control.

### 4.3. DNA Extraction

Cases with sufficient FFPE tissue were subjected to molecular testing. Representative FFPE blocks were used for DNA extraction. Four to six 10 μm-sections on glass slides were used for each case based on tissue size. DNA extraction was performed using QIAamp DNA FFPE tissue kit (Qiagen, Germantown, MD, USA) based on the manufacturer’s instruction. In brief, the tumor region on section was applied with lysis buffer, scraped off the glass slide, and collected into a microcentrifuge tube in a total volume of 180 μL. The sample was then incubated with 20 μL proteinase K at 56 °C for 1 h and then mixed with 200 μL buffer AL and 200 μL ethanol. The mixture was then transferred to the MinElute column, centrifuged to remove the buffer, and passed sequentially by buffer AW1 and AW2. Finally, 60 μL of buffer ATE was added to the column and the eluate containing extracted DNA was collected. 

### 4.4. Targeted Next-Generation Sequencing (NGS)

We performed targeted NGS panel to identify mutation at *BRAF* exon 15 (NM_004333.4), *RAS* codon 12, 13, and 61 (*H-RAS* NM_005343.2, *N-RAS* NM_002524.4, and *K-RAS* NM_033360.2) [36], *POLE* exonuclease domain encompassing codons 268 to 471 (NM_006231.2) [37], *TERT* promoter (NC_000005.9) [12], and *TP53* whole coding region (NM_000546.5). The amount of 250 ng tumor genomic DNA of each individual was used for library construction of the targeted gene regions using QIAseq target DNA system (Qiagen). Briefly, DNA was enzymatically fragmented and end-repaired in a 25 μL reaction containing 2.5 μL 10× fragmentation buffer and 5 μL fragmentation enzyme mix. The reaction was carried out at 4 °C for 1 min, 32 °C for 24 min, and 65 °C for 30 min. Immediately after the reaction, 10 μL 5× ligation buffer, 5 μL DNA ligase, 2.8 μL 25 μM barcoded adapters, and water were added to reach a total volume at 50 μL. The reaction was incubated at 20 °C for 15 min. To ensure complete removal of free barcoded adapters, each reaction was purified for two rounds using beads. To amplify the barcoded DNA fragments, the purified DNA was then mixed with 10 nM each of target primer (a total of 72 target specific primers), 400 nM Forward primer, 1× PCR buffer, and 0.8 μL HotStarTaq DNA polymerase in a 20 μL reaction. The PCR was carried with the following conditions: 95 °C for 13 min, 98 °C for 2 min; six cycles of 98 °C for 15 s and 65 °C for 15 min; 72 °C for 5 min. The reaction was then incubated with beads to remove remaining excess primers. The DNA product was subjected to another round of amplification by mixing with 400 nM Universal primer, 400 nM Index primer, 1X PCR buffer, and 1 μL HotStarTaq DNA polymerase in a volume of 20 μL, with the following PCR conditions: 95 °C for 13 min, 98 °C for 2 min, 20 cycles of 98 °C for 15 s and 60 °C for 2 min; 72 °C for 5 min. The DNA library was purified by incubation with beads, and the final pool subjected to targeted sequencing using Illumina MiniSeq sequencer (Illumina, San Diego, CA, USA). The raw outputs of each case were>16 Mb, and the average depth of target regions was more than 500 folds. The sequence of each read was trimmed based on the quality score (Q30), and reads with length less than 45 bp were discarded in subsequent analysis. Reads were aligned to the human hg19 reference genome using BWA-MEM [38]. GATK Unified Genotyper was used for variant calling [39]. Variant Effect Predictor was used to annotating the variants [40]. Variants with an allelic frequency ≤5% were excluded. Only non-synonymous mutations reported in the Sanger Institute Catalogue were taken into account.

### 4.5. Mass Spectrometry for PI3KCA Mutations

Analysis of hot spot somatic mutations of *PI3KCA* in exon 9 and exon 20 (NM_006218.4) [41] was performed using Agena MassARRAY platform with iPLEX reagent chemistry (Agena, San Diego, CA, USA). The genotypes of the codons assayed are listed in Supplementary Table S3. The specific PCR primer and extension primer sequences were designed with Assay Designer software package (v.4.0). Multiplex PCR reaction was carried by mixing 10 ng genomic DNA sample, 1 U Taq polymerase, 500 nmol each of PCR primers, and 2.5 mM dNTP in 5 μL reaction volume and amplified in a thermocycler for 45 cycles. Unincorporated dNTPs were deactivated using 0.3 U of shrimp alkaline phosphatase. Single base extension reaction was performed by adding a mix of specific extension primers, dideoxynucleotide terminators, and extension enzyme, followed by thermocycling according to manufacturer’s instruction. The specific extension primers anneal directly to the mutation sites, and were extended and terminated by the dideoxynucleotide terminators complementary to the target somatic mutational site. The reaction was purified by incubation with cation exchange resin, and 7 μL of the purified product was loaded onto a matrix pad of a SpectroCHIP where the analyte crystalizes with a pre-spotted MALDI matrix. SpectroCHIPs were loaded in MassARRAY Analyzer 4 where ionization and signal detection were carried, and base calling reported by clustering analysis with TYPER 4.0 software.

### 4.6. Statistical Analysis

Categorical data were analyzed using Fisher exact test and logistic regression for univariate and multivariate analyses. Survival curves were built using the Kaplan–Meier method, and log-rank test was applied to test for differences between the variables. The *p*-values were two-sided, and a value <0.05 was considered significant. All analyses were performed using R software (version 3.5.1, R Foundation for Statistical Computing, Vienna, Austria).

## 5. Conclusions

This study illustrates clinicopathological features in ATC stratified by *BRAF*^V600E^ and *RAS* mutations. Importantly, *RAS* and *PIK3CA* mutations were found to be negative prognostic indicators in ATC. In concordance with previous reports, we demonstrated that genetic hallmarks in ATC including *TERT* promoter and *TP53* mutations were prevalent in ATC. MMR-D or *POLE* exonuclease domain mutations were not identified in our study cohort. Acknowledging molecular profiles of ATC may have clinical implications regarding emerging targeted therapies, including BRAF, RAS, and PI3 kinase inhibitors.

## Figures and Tables

**Table 1:** Summary of clinicopathological features of 39 anaplastic thyroid carcinoma cases.

**Age, n (%)**		
≤55	4 (10.3)	
>55	35 (89.7)	
**Gender, n (%)**		
Female	20 (51.3)	
Male	19 (48.7)	
**Stage, n (%) ^a^**		
IVA	5 (13.9)	
IVB	11 (30.6)	
IVC	20 (55.6)	
**Surgery, n (%)**		
Subtotal thyroidectomy	9 (23.1)	
Total thyroidectomy	24 (61.5)	
Biopsy only	6 (15.4)	
**Other Therapies ^b^, n (%)**		
Chemotherapy	7 (24.1)	
Radioiodine	6 (21.4)	
External radiation	7 (25)	
Targeted therapy	5 (15.1)	
Tumor size ^c^ (cm)	7.14	
**Histologic Pattern, n (%)**		
Epithelial	+	28 (71.8%)
	-	11 (28.2%)
Giant cell	+	5 (12.8%)
	-	34 (87.2%)
Sarcomatoid	+	17 (43.6%)
	-	22 (56.4%)
**Coexisting WDTC, n (%)**		
PTC	+	10 (25.6)
	-	29 (74.4)
FTC	+	3 (7.7)
	-	36 (92.3)

^a^ Of patients with available information regarding tumor stage. ^b^ Of patients with available information regarding therapies. ^c^ Median size of the tumors. WDTC, well-differentiated thyroid carcinoma; PTC, papillary thyroid carcinoma; FTC, follicular thyroid carcinoma.

**Table 2:** Summary of the results of immunohistochemistry.

	Case Number	n (%)			
		PAX8	TTF-1	BRAF VE1	Retained MMR Proteins
**All**	39	22 (56.4)	2 (5.1)	10 (25.6)	39 (100)
**Coexisting WDTC**					
**None**	26	15 (5.8)	2 (7.7)	4 (15.4)	26 (100)
**PTC**	10	7 (70)	0	6 (60)	10 (100)
**FTC**	3	0	0	0	3 (100)

WDTC, well-differentiated thyroid carcinoma; PTC, papillary thyroid carcinoma; FTC, follicular thyroid carcinoma; MMR, mismatch repair.

**Table 3:** Summary of clinicopathological features of 27 anaplastic thyroid carcinoma cases with stratification by *BRAF*_V600E_ and *RAS* mutations.

		*BRAF*	*RAS*	Non-*BRAF/RAS*	*p* Value
**Case Number**		7	11	9	
**Age, n (%)**					
≤55		1 (14.3)	1 (9.1)	2 (22.2)	0.803
>55		6 (85.7)	10 (90.9)	7 (77.8)	
**Gender, n (%)**					
Female		2 (28.6)	10 (90.9)	4 (44.4)	0.012 *
Male		5 (71.4)	1 (9.1)	5 (55.6)	
**Stage, n (%) ^a^**					
IVA		1 (14.3)	0 (0)	2 (25)	0.445
IVB		3 (42.9)	4 (44.4)	1 (12.5)	
IVC		3 (42.9)	5 (55.6)	5 (50)	
**Surgery, n (%)**					
Subtotal Tx		1 (14.3)	2 (18.2)	4 (44.4)	0.488
Total Tx		6 (85.7)	8 (72.7)	4 (44.4)	
Biopsy only		0	1 (9.1)	1 (11.1)	
**Other Therapies ^b^, n (%)**					
Chemotherapy		1 (16.7)	3 (33.3)	2 (33.3)	0.851
Radioiodine		1 (16.7)	2 (22.2)	0 (0)	0.763
External radiation		1 (16.7)	4 (44.4)	1 (20)	0.568
Targeted therapy		2 (33.3)	1 (10)	0 (0)	0.314
Tumor size, cm		5.1	7.8	7.5	0.253
**Histologic Pattern, n (%)**					
Epithelial	+	6 (85.7)	8 (72.7)	6 (66.7)	0.860
	-	1(14.3)	3 (27.3)	3 (33.3)	
Giant cell	+	1 (14.3)	1 (9.1)	1 (11.1)	1
	-	6 (85.7)	10 (90.9)	8 (88.9)	
Sarcomatoid	+	1 (14.3)	5 (45.5)	7 (77.8)	0.045 *
	-	6 (85.7)	6 (54.5)	2 (22.2)	
**Coexisting WDTC, n (%)**					
PTC	+	3 (42.9)	2 (18.2)	1 (11.1)	0.369
	-	4 (57.1)	9 (81.8)	8 (88.9)	
FTC	+	0 (0)	1 (9.1)	1 (11.1)	1.000
	-	7 (100)	10 (90.9)	8 (88.9)	

^a^ Of patients with available information regarding tumor stage. ^b^ Of patients with available information regarding therapies * Statistically significant. Tx, thyroidectomy; WDTC, well-differentiated thyroid carcinoma; PTC, papillary thyroid carcinoma; FTC, follicular thyroid carcinoma.

## Supplementary Materials

The following are available online at https://www.mdpi.com/2072-6694/12/7/1973/s1, Supplementary data 1: Mutational profile of anaplastic thyroid carcinoma cases detected by next-generation sequencing, Supplemental data 2: PIK3CA mutations in anaplastic thyroid carcinoma cases detected by mass spectrometry, Table S1: Clinicopathological features of RAS-mutated and RAS wild-type ATC cases, Table S2: Clinicopathological features of ATC cases wild type for RAS and BRAFV600E and cases with either one mutation, Table S3: List of PIK3CA mass spectrometry assay panel.
