# Supplementary material for: Characterization of Driver Mutations in Anaplastic Thyroid Carcinoma Identifies RAS and PIK3CA Mutations as Negative Survival Predictors
Source: Cancers (Basel). 2020 Jul 20;12(7):1973. doi: 10.3390/cancers12071973 (PMC7409295; doi:10.3390/cancers12071973)
Supplement: Supplementary file 1 [file cancers-12-01973-s001.pdf]

Supplementary Data 1. Mutational profile of anaplastic thyroid carcinoma cases detected by next-generation sequencing.

Case No.	Gene	Variant	Alternative Variant Frequency	Chromosome	Coordinate	CDS Position	Protein Position	Amino Acids
1	BRAF	A>A/T	21.94	7	140453136	1799	600	V/E
1	TERT C228T	G>G/A	22.58	5	1295228			
1	TP53	C>C/A	27.08	17	7578403	527	176	C/F
2	BRAF	A>A/T	39.31	7	140453136	1799	600	V/E
2	TERT C228T	G>G/A	32.53	5	1295228			
2	TP53	A>A/C	33.56	17	7578393	537	179	H/Q
3	BRAF	A>A/T	19.5	7	140453136	1799	600	V/E
3	TERT C228T	G>G/A	26.76	5	1295228			
3	TP53	G>G/A	26.22	17	7578212	637	213	R/*
4	BRAF	A>A/T	37.17	7	140453136	1799	600	V/E
4	TERT C228T	G>G/A	26.73	5	1295228			
5	BRAF	A>A/T	15.79	7	140453136	1799	600	V/E
5	TERT C228T	G>G/A	16.22	5	1295228			
6	BRAF	A>A/T	29.77	7	140453136	1799	600	V/E
6	TERT C228T	G>G/A	28.42	5	1295228			
7	BRAF	A>A/T	34.21	7	140453136	1799	600	V/E
8	KRAS	T>T/C	54.58	12	25380276	182	61	Q/R
8	TERT C228T	G>G/A	44.28	5	1295228			
8	TP53	T>T/C	63.7	17	7578271	578	193	H/R
9	KRAS	C>C/G	52.57	12	25398284	35	12	G/A
9	TERT C228T	G>G/A	9.8	5	1295228		0	
9	TP53	G>G/A	23.9	17	7577121	817	273	R/C
10	KRAS	T>T/C	20.78	12	25380276	182	61	Q/R
10	TP53	CA>CA/C	13.33	17	7579345	341	114	L/X
10	TP53	G>G/A	14.74	17	7577121	817	273	R/C
11	NRAS	G>G/T	54.89	1	115256530	181	61	Q/K
11	TERT C228T	G>G/A	38.56	5	1295228		0	
11	TP53	G>G/T	44.44	17	7578384	546	182	C/*
12	NRAS	T>T/C	52.67	1	115256529	182	61	Q/R
12	TERT C228T	G>G/A	34.33	5	1295228		0	
12	TP53	C>C/G	35.69	17	7578534	396	132	K/N
13	NRAS	T>T/C	56	1	115256529	182	61	Q/R
13	TERT C228T	G>G/A	23.3	5	1295228		0	

13	TP53	G>G/A	30.32	17	7578272	577	193	H/Y
14	NRAS	T>T/C	7.59	1	115256529	182	61	Q/R
14	TERT C228T	G>G/A	7.6	5	1295228		0	
14	TP53	G>G/C	5.18	17	7578464	466	156	R/G
15	NRAS	T>T/C	41	1	115256529	182	61	Q/R
15	TERT C228T	G>G/A	10.46	5	1295228		0	
16	NRAS	C>C/T	60.71	1	115258747	35	12	G/D
16	TERT C228T	G>G/A	28.91	5	1295228		0	
17	NRAS	T>T/C	24.24	1	115256529	182	61	Q/R
17	TP53	G>G/C	30	17	7577539	742	248	R/G
18	NRAS	T>T/C	43.64	1	115256529	182	61	Q/R
18	TP53	A>A/C	31.73	17	7578268	581	194	L/R
19	TERT C228T	G>G/A	25.64	5	1295228		0	
19	TP53	G>G/A	44.47	17	7577532	749	250	P/L
20	TERT C228T	G>G/A	74.65	5	1295228		0	
20	TP53	A>A/G	65.34	17	7577129	809	270	F/S
21	TERT C228T	G>G/A	26.38	5	1295228		0	
21	TP53	G>G/C	52.62	17	7577512	769	257	L/V
22	TERT C228T	G>G/A	43.9	5	1295228		0	
22	TP53	T>T/A	64.5	17	7578266	583	195	I/F
23	TERT C228T	G>G/A	24.28	5	1295228		0	
23	TP53	TG>TG/T	45.07	17	7576896	949	317	Q/X
24	TERT C228T	G>G/A	15.34	5	1295228		0	
24	TP53	G>G/C	14.07	17	7578525	405	135	C/W
25	TERT C228T	G>G/A	44.34	5	1295228		0	
26	TERT C228T	G>G/A	17.65	5	1295228		0	
27	TP53	G>G/A	9.86	17	7578212	637	213	R/*

Supplemental Data 2. *PIK3CA* mutations in anaplastic thyroid carcinoma cases detected by mass spectrometry

Case No.	cDNA_Mutation	Amino_Acid_Change
5	PIK3CA c.3140A>G	p.His1047Arg
6	PIK3CA c.3140A>G	p.His1047Arg
10	PIK3CA c.1624G>A	p.Glu542Lys
22	PIK3CA c.1633G>A	p.Glu545Lys

Supplementary Table S1. Clinicopathological features of *RAS*-mutated and *RAS* wild-type anaplastic thyroid carcinoma cases

		RAS	WT	Univariate			Multivariate		
				OR	95% CI	p-value	OR	95% CI	p-value
**Age, n**									
>55		10	13	2.31	0.25-50.65	0.496	1.06	0.03-57.07	0.973
≤55		1	3	1			1		
**Gender, n**									
Female		10	6	16.67	2.33-347.59	0.016*	20.31	2.24-571.25	0.022*
Male		1	10	1			1		
**Stage, n**[a]									
IVC		5	8	1.09	0.21-6.03	0.916	0.77	0.07-8.10	0.827
IVA or IVB		4	7	1			1		
**TTx**	**+**	8	10	1.6	0.31-9.53	0.581	1.49	0.13-19.70	0.744
	**-**	3	6	1			1		
**Sarcomatoid pattern**	**+**	5	8	0.83	0.17-3.92	0.816	0.54	0.04-5.49	0.606
	**-**	6	8	1			1		
**PTC**	**+**	2	4	0.67	0.08-4.25	0.677	0.61	0.04-8.12	0.710
	**-**	9	12	1			1		

[a]Of patients with available information regarding tumor stage. *Statistically significant. ATC, anaplastic thyroid carcinoma; TTx, total thyroidectomy, PTC, papillary thyroid carcinoma; OR, odds ratio; CI, confidence interval.

Supplementary Table S2. Clinicopathological features of anaplastic thyroid carcinoma cases wild type for *RAS* and *BRA*^{FV600E} and cases with either one mutation

		Non-B/R	Mut	Univariate			Multivariate		
				OR	95% CI	p-value	OR	95% CI	p-value
**Age, n**									
>55		7	16	0.44	0.04-4.25	0.451	0.08	0-3.23	0.220
≤55		2	2	1			1		
**Gender, n**									
Female		4	12	0.40	0.07-2.04	0.273	0.09	0-1.21	0.125
Male		5	6	1			1		
**Stage, n**[a]									
IVC		5	8	1.67	0.30-10.49	0.564	1.27	0.05-41.67	0.881
IVA or IVB		3	8	1			1		
**TTx**	+	4	14	0.23	0.04-1.24	0.093	0.17	0.01-1.77	0.176
	-	5	4	1			1		
**Sarcomatoid pattern**	+	7	6	7	1.25-57.94	0.040*	23.17	1.24-2424.4	0.089
	-	2	12	1			1		
**PTC**	+	1	5	0.33	0.02-2.53	0.342	0.21	0-6.91	0.416
	-	8	13	1			1		

[a]Of patients with available information regarding tumor stage. *Statistically significant. ATC, anaplastic thyroid carcinoma; Non-B/R: cases without *BRAF*^{V600E} and *RAS* mutations; Mut, cases with either *BRAF*^{V600E} or *RAS* mutations; TTX, total thyroidectomy; PTC, papillary thyroid carcinoma; OR, odds ratio; CI, confidence interval.

Supplementary Table S3. List of *PIK3CA* mass spectrometry assay panel

Exon	Codon	Amino acid mutation	Coding sequence mutation
9	542	p.E542K	c.1624G>A
	545	p.E545K	c.1633G>A
		p.E545Q	c.1633G>C
		p.E545G	c.1634A>G
		p.E545V	c.1634A>T
	546	p.Q546E	c.1636C>G
		p.Q546K	c.1636C>A
		p.Q546L	c.1637A>T
		p.Q546P	c.1637A>C
		p.Q546R	c.1637A>G
	549	p.D549N	c.1645G>A
20	1047	p.H1047L	c.3140A>T
		p.H1047R	c.3140A>G
